# RNA Sequencing for Personalized Treatment of Metastatic Leiomyosarcoma: Case Report

**DOI:** 10.3389/fonc.2021.666001

**Published:** 2021-08-30

**Authors:** Alexander Seryakov, Zaynab Magomedova, Maria Suntsova, Anastasia Prokofieva, Elizaveta Rabushko, Alexander Glusker, Lyudmila Makovskaia, Marianna Zolotovskaia, Anton Buzdin, Maxim Sorokin

**Affiliations:** ^1^Medical Holding SM-Clinic, Moscow, Russia; ^2^The Laboratory of Clinical and Genomic Bioinformatics, I.M. Sechenov First Moscow State Medical University, Moscow, Russia; ^3^World-Class Research Center “Digital Biodesign and Personalized Healthcare”, Sechenov First Moscow State Medical University, Moscow, Russia; ^4^Faculty of Fundamental Medicine, Lomonosov Moscow State University, Moscow, Russia; ^5^Laboratory of Translational Genomic Bioinformatics, Moscow Institute of Physics and Technology, Dolgoprudny, Russia; ^6^Shemyakin-Ovchinnikov Institute of Bioorganic Chemistry, Russian Academy of Sciences, Moscow, Russia; ^7^OmicsWay Corp, Walnut, CA, United States

**Keywords:** uterine leiomyosarcoma, regorafenib, RNA sequencing, cancer gene fusion, personalized therapy, oncobox, whole-exome sequencing, targeted therapeutics

## Abstract

Uterine leiomyosarcoma (UL) is a rare malignant tumor that develops from the uterine smooth muscle tissue. Due to the low frequency and lack of sufficient data from clinical trials there is currently no effective treatment that is routinely accepted for UL. Here we report a case of a 65-years-old female patient with metastatic UL, who progressed on ifosfamide and doxorubicin therapy and developed severe hypertensive crisis after administration of second line pazopanib, which lead to treatment termination. Rapid progression of the tumor stressed the need for the alternative treatment options. We performed RNA sequencing and whole exome sequencing profiling of the patient’s biopsy and applied Oncobox bioinformatic algorithm to prioritize targeted therapeutics. No clinically relevant mutations associated with drug efficiencies were found, but the Oncobox transcriptome analysis predicted regorafenib as the most effective targeted treatment option. Regorafenib administration resulted in a complete metabolic response which lasted for 10 months. In addition, RNA sequencing analysis revealed a novel cancer fusion transcript of *YWHAE* gene with fusion partner *JAZF1.* Several chimeric transcripts for *YWHAE* and *JAZF1* genes were previously found in uterine neoplasms and some of them were associated with tumor prognosis. However, their combination was detected in this study for the first time. Taken together, these findings evidence that RNA sequencing may complement analysis of clinically relevant mutations and enhance management of oncological patients by suggesting putative treatment options.

## Background

Uterine leiomyosarcoma (UL) is the most common type of uterine sarcomas which accounts for about 1-2% of uterine malignancies (1, 2). This tumor has a higher rate of metastasis without prior lymph node involvement compared to adenocarcinomas (2). Most patients experience vaginal bleeding, as well as in cases of patients with adenocarcinomas. Other patients may experience local discomfort from uterine enlargement. The average age of UL diagnosis is about 50 years (3). But in the early stages, the disease course is usually asymptomatic and is often mistakenly diagnosed as uterine leiomyoma (3).

Approximately 0.5% of patients undergoing a hysterectomy for a suspected benign leiomyoma then demonstrate UL, and it is problematic to distinguish between the two tumors before surgery (2). Moreover, since laparoscopic extraction, including morcellation, there is a risk of spreading latent UL to the entire abdominal cavity (2).

Leiomyosarcomas are thought to occur independently of leiomyoma (4). These tumors are characterized by abundant mitoses, prominent cellular atypia, and necrosis. The coincidence of two of the three signs indicates a risk of metastasis of more than 10% (4). When leiomyoma and UL cannot be differentiated, the term STUMP (smooth tumors of undefined malignant potential) is used for diagnosis (5). The lungs are the most frequent site of metastasis. Thus, the initial assessment should include a chest CT scan (6).

Due to the lack of data from randomized trials, management tactics vary. The initial treatment is surgical intervention by hysterectomy. Lymph node involvement is rare, and usually lymph node dissection is not required (7). The use of radiation therapy showed no difference in either overall survival or progression-free survival, and did not lead to an improvement in local control (the rate of local relapses in the radiation therapy group was 20% compared to 24% in the case of surgical treatment in a randomized phase III EORTC study) (8).

For UL, there is no effective chemotherapy scheme today. The best results were shown for the following regimens: (*i*) gemcitabine and docetaxel combination in advanced setting. Frequency of the objective response to this regimen was 36% (9); (*ii*) paclitaxel, with partial or complete response rate of ~8% of the cases (10); (*iii*) doxorubicin monotherapy with 15% response rate (11); (*iv*) doxorubicin and ifosfamide treatment results in moderate response rates of 10%-30% (12). Trabectedin and pazopanib may be further used as the second line treatment of UL. Trabectedin treatment results in 1-year survival rate of 61% (13). In turn, administration of pazopanib allows to achieve long-term stabilization or partial regression of the tumor and allows to increase the median survival rate to 17.5 months (14).

Thus, to date there is no effective standard treatment for UL, and personalized approach may be needed for better patient management. Such an approach may be based on high-throughput gene expression profiling, because no clinically actionable mutations were described for UL in the literature. Gene expression profiling using RNA sequencing and further bioinformatic analysis may, in turn, provide insights on the pathological processes altered in a specific tumor (15). The only genetic test utilizing both DNA analysis for mutation profiling and RNA analysis for gene expression profiling is Oncobox (16). Oncobox uses advanced pathway analysis of gene expression data to build personalized rating of targeted drugs (17). Here we describe a case of successful application of the Oncobox testing to select treatment for metastatic UL.

## Case Presentation

**Figure 1:**
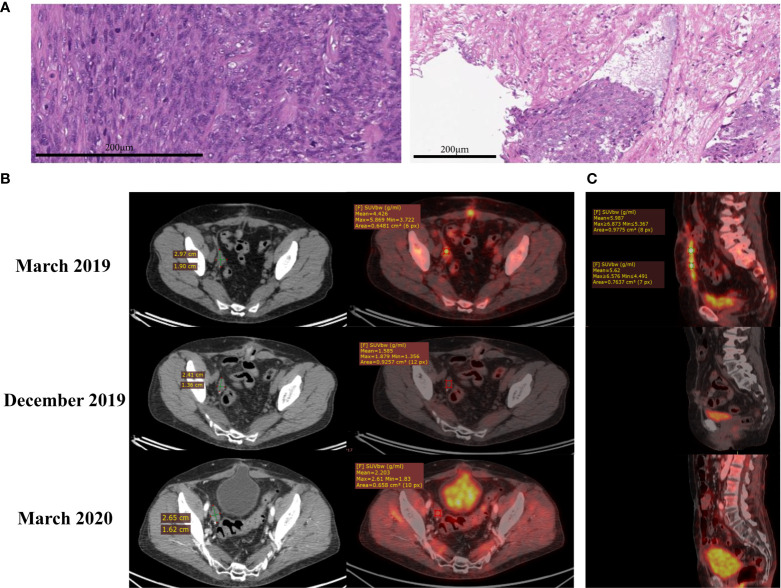
**(A)** Hematoxylin and eosin (H&E) staining shows uterine leiomyosarcoma (left) with lymphovascular invasion (right). **(B)** CT (left) and PET-CT (right) scans of the pelvis. Lesion in the area of the removed right ovary in March 2019, December 2019 and March 2020. **(C)** PET-CT scans of the abdomen. Nodes along the anterior abdominal wall in March 2019, December 2019 and March 2020, sagittal plane.

In February 2019, a 65-year-old woman underwent uterine extirpation from the upper third of the vagina for uterine fibroids, which had been diagnosed for 18 years. Histological examination of the material indicated UL (**Figure 1A**), G1, pT1b, size 12 cm, with invasion of the entire thickness of the uterine myometrium wall and endometrial invasion. IHC examination revealed spindle-shaped cells with mild nuclear polymorphism and an abundance of mitoses (**Figure1A**). Lymphovascular invasion was detected (**Figure 1A**), resection margin and appendages were without a tumor. A histological examination of the omentum was also performed, and no signs of tumor growth were found.

In March 2019, PET-CT revealed lesions in the lungs (size 7 mm and 11 mm, **Figure S1**), in the area of the removed right ovary (3 cm, **Figure 1B**) and along the anterior abdominal wall (up to 1 cm^2^, **Figure 1C**). First-line polychemotherapy (PCT) with ifosfamide (2500 mg/m2/day on days 1 to 4) and doxorubicin (25 mg/m2 intravenously, on days 1 to 3) started, and the patient received two out of four prescribed therapy courses. The patient developed fibril neutropenia and grade 2 thrombocytopenia; therefore, the dose was reduced for the next course to 1875 mg/m2/day intravenously, on days 1 to 4 for ifosfamide and to 18.7 mg/m2 intravenously, on days 1 to 3 for doxorubicin. According to MRI data from June 2019 (**Figure S2**), the disease progressed: a pathological lesion with dimensions - transverse 2.4 cm, vertical 3.0 cm, craniocaudal 7.5 cm, unevenly accumulating a contrast agent was observed on the right, with the spread to the area of the iliac vessels.

Since the disease progressed after the PCT, the patient received monotherapy with targeted tyrosine kinase inhibitor pazopanib (800 mg daily) as the second line in June. However, the treatment was terminated after the second dose (second day of the treatment) due to the development of a severe hypertensive crisis. MRI in September 2019 revealed lesions in the right iliac region - secondary altered lymph nodes, with signs of invasion in the right ureter (**Figure S3**).

As the patient rapidly progressed on standard treatment, an attempt was made to find an alternative treatment option. To identify third-line therapy, Oncobox molecular diagnostic test was performed for the patient tumor biopsy specimen obtained during the operation in February 2019 containing 95% tumor cells. Oncobox test used included whole-exome sequencing (WES) and RNA sequencing (RNAseq) of tumor biosample. WES data are used to identify diagnostic mutations and to calculate tumor mutation burden, whereas RNAseq information helps identifying molecular drug targets that are differentially expressed in the tumor, and also differentially regulated molecular pathways compared to the healthy tissues (16, 18). Annotated Oncobox pathway database was recently published (19). The healthy control tissues were sequenced previously by Oncobox (20) using the same equipment and protocols. Based on the drug target and molecular pathway information, Oncobox algorithm returns personalized rating of targeted therapeutics (17). To this end, *balanced efficiency score* (BES) of each targeted cancer drug is calculated that is based on the extent of up/downregulation of drug target genes and drug target pathways (15). The latter complements mutation data and helps identifying possible treatment options even when no clinically actionable mutations can be found. This approach was found effective for advanced solid tumors in several clinical screens (21–24) and trials (25, 26), and used for off-label drug prescriptions in the progressive tumors (27–31).

In the present case, whole exome sequencing (WES) and RNA sequencing (RNAseq) profiling of tumor sample was performed followed by bioinformatic analysis (17). The tumor sample showed no signs of microsatellite instability according to (32). WES identified 583 non-synonymous mutations in protein-coding genes, and an overall tumor mutation burden value (calculated also including synonymous mutations according to (33) was 9.8 per megabase which couldn’t support using immune checkpoint inhibitors according to (34). No clinically actionable mutations were identified in genes *ATRX, BRCA1, BRCA2, ATM, BARD1, BRIP1, CHEK1, CHEK2, FAM175A, MRE11A, NBN, PALB2, RAD51B, RAD51C, RAD51D, RAD54L*, thus platinum compounds and PARP inhibitors would potentially be ineffective (35, 36). There were no activating mutations in the *BRAF, EGFR*, and *PIK3CA* genes and no amplification was found for *ERBB2* (*HER2*), thus corresponding inhibitors could not be prescribed.

**Figure 2:**
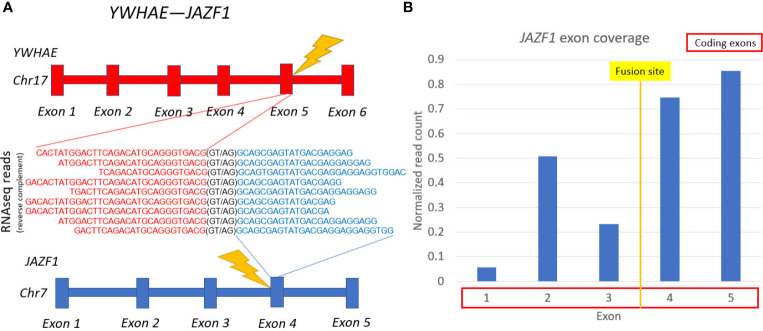
Schematic representation of YWHAE-JAZF1 fusion transcript identified. **(A)** gene structures upstream and downstream of fusion site. **(B)** JAZF1 gene exon coverage by normalized RNA sequencing reads.

However, RNAseq analysis detected a previously unknown in-frame cancer fusion transcript of genes *YWHAE* and *JAZF1*, directly supported by nine sequencing reads (**Figure 2**). The fifth exon of *YWHAE* was fused with the fourth exon of *JAZF1*. *JAZF1* is frequently amplified and overexpressed in many cancers (37). The relative exon coverage of *JAZF1* by RNAseq reads downstream to the fusion site was higher than upstream, thus confirming abnormal activation of this gene (**Figure 2B**). Other fusions separately involving either *YWHAE* or *JAZF1* were previously reported for uterine sarcomas (38), some of them (*YWHAE* with fusion partners *NUTM2A* and *NUTM2B*) were associated with poor prognosis (39). However, *YWHAE*-*JAZF1* combination, to our knowledge, was identified here for the first time, and it could not drive a decision on the patient management.

Gene Ontology (GO) analysis of the top-100 up-regulated genes in the patient’s biosample according to RNAseq data revealed terms associated with extracellular matrix organization, mesenchyme development and organ development (**Figure S4**). GO-analysis of the 100 most down-regulated genes revealed terms associated with muscle system process, actin−mediated cell contraction, and sarcomere organization. Fold changes for all 36596 genes analyzed can be found in **Table S1**.

**Figure 3:**
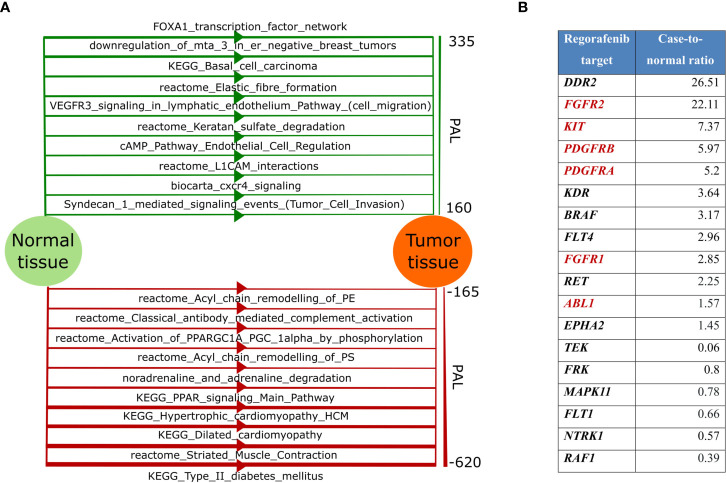
**(A)** Top-10 up-regulated (green color, top) and top-10 down-regulated (red color, bottom) molecular pathways in the patient’s tumor. Line width for each pathway is proportional to the pathway activation level (PAL), scale for PAL values is presented on the right; **(B)** Gene expression level of Regorafenib targets. Targets included in the “KEGG Pathways in cancer” pathway are highlighted in red.

We then used RNAseq data to analyze molecular pathways with altered cancer-to-healthy tissue activation profiles using Oncobox algorithm (21, 40, 41). We found that the most strongly *upregulated* pathways dealt with (*i*) *FOXA1 transcription factor* network, (*ii*) *downregulation of MTA3 in breast cancer*, (*iii*) *basal cell carcinoma* network, (*iv*) *elastic fiber formation*, (*v*) *cell migration* branch of VEGFR signaling in lymphatic endothelium pathway, (*vi*) *keratan sulfate degradation*, (*vii*) *endothelial cell regulation* branch of cAMP pathway, (*viii*) *L1CAM interactions*, (*ix*) *CXCR4 signaling*, and (*x*) *tumor cell invasion* branch of Syndecan 1 signaling pathway (**Figure 3A**, top). The main *downregulated* pathways dealt with (*i*) *type 2 diabetes* network, (*ii*) *muscle contraction*, (*iii-iv*) *cardiomyopathy* network, (*v* and *viii*) *PPAR signaling*, (*vi*) *noradrenaline and adrenaline degradation*, (*vii* and *x*) acyl chain remodeling, and (*ix*) *antibody-mediated complement activation* (**Figure 3A**, bottom).

Applying Oncobox algorithm to RNAseq data, we also built personalized rating of 159 cancer drugs with 164 molecular targets (17). According to the Oncobox report, the patient’s tumor was predicted to be sensitive to the following top-10 targeted drugs (in a decreasing efficiency order): *(i) regorafenib*, *(ii) lenvatinib*, (*iii*) *nintedanib*, *(iv) dovitinib*, *(v) tivozanib*, (*vi*) *dasatinib*, (*vii*) *sunitinib*, (*viii*) *sorafenib*, (*ix*) *pazopanib*, and (*x*) *midostaurin* (**Table S1**).

Based on the results of this molecular profiling, the institutional tumor board approved administration of regorafenib, a targeted tyrosine kinase inhibitor with multiple specificities (**Figure 3B**). The patient received regorafenib as monotherapy (80 mg daily) from September till December 2019, and PET-CT investigation from 21.12.2019 revealed a complete metabolic regression of the tumor (**Figures 1** and **S1**). Regorafenib administration was then continued till February 2020 in the same regimen. Thus, the patient received 5 courses of regorafenib in total. Complete metabolic response of the patient’s tumor was confirmed in March 2020 (**Figures 1B, C**), but then the treatment was terminated due to poor tolerability of the drug: the patient developed gastrointestinal toxicity, stomatitis, cheilitis, and arterial hypertension. The tumor progressed in July 2020: pathological lesions accumulating contrast agents were found in the lungs, omentum and peritoneum (**Figures S5** and **S6**). Regorafenib treatment was resumed, and the tumor partially regressed in September 2020. However, same regorafenib side effects occurred, and it was decided to make a second attempt of pazopanib treatment. The patient received pazopanib from September till December 2020, but the disease progressed, and the patient had moderate anemia of chronic diseases. It was decided to administer regorafenib and partial response was documented in April 2021. As for August 2021 the patient is alive, receives regorafenib therapy and has mild anemia of chronic diseases.

## Discussion

Regorafenib inhibits multiple tyrosine kinases involved in tumor angiogenesis (*VEGFR1-3, TIE2*), oncogenic transformation (*KIT, RET, RAF1, BRAF*), and shaping tumor microenvironment (*PDGFR*, *FGFR*) (42). *KIT* is a proto-oncogene encoding receptor tyrosine kinase. When bound to its ligands, it phosphorylates and activates the PI3K/AKT signaling axis. Previously strong diffused expression of *KIT* was observed in ~75% of leiomyosarcomas (43), and in 53% of uterine leiomyosarcomas (44). Genes *PDGFRA* and *PDGFRB* encode the platelet-derived growth factor receptors that play a significant role in cell growth and differentiation. Previously expression changes of *PDGFRA* and *PDGFRB* in UL have not been sufficiently investigated with the exception of a single study showing that their expression was increased in ~60% of UL (45). *FGFR1* encodes one of the fibroblast growth factor receptors. Binding FGFR1 to its ligand also leads to the activation of PI3K/AKT axis. This gene upregulation was previously observed in several UL cell lines, and its targeted inhibition resulted in strong suppression of cell proliferation and survival (46). In turn, the PI3K/AKT axis makes a significant contribution to the development of leiomyosarcomas, and this pathway inhibition leads to suppression of growth and activation of apoptosis on both *in vitro* and *in vivo* UL models (47).

**Figure 4:**
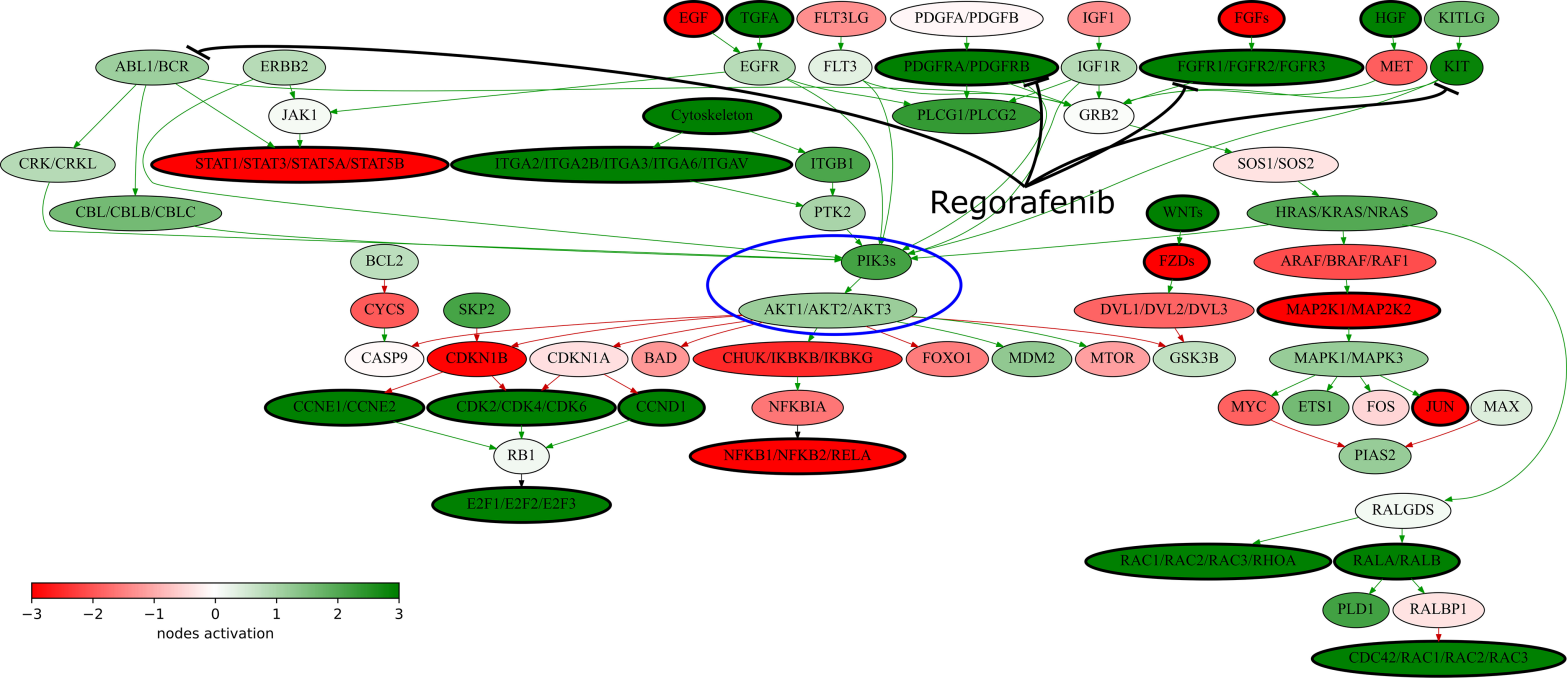
“KEGG Pathways in cancer” signaling pathway shown as an interacting network. This pathway was hyperactivated in the patient’s tumor tissue. Green arrows indicate activation, red arrows–inhibition. Transcript nodes are shown in ovals. The color depth of transcript nodes reflects the extent of node activation (logarithms of the case-to-normal (CNR) expression rate for each node, in which “normal” is a geometric average between expression levels in normal tissue samples). Molecular targets of regorafenib are indicated by black arrows. Visualization was implemented using Oncobox software. The PI3Ks-AKT signaling axis is marked in blue ellipse.

RNAseq analysis revealed twelve upregulated versus only six downregulated regorafenib target genes in the patient’s tumor (**Figure 3B**). Moreover, multiple signaling pathways containing regorafenib targets were upregulated in the tumor according to Oncobox analysis (**Table S1**), For example, all regorafenib targets included in “KEGG Pathways in cancer” network show increased expression levels (**Figure 4**). The *PDGFRA-B*, *FGFR1-2*, and *KIT* gene products activate the PI3K-AKT axis (**Figure 4**, circled), which, in turn, was also activated. Thus, increased expression levels of multiple targeted gene products along with the upregulated targeted signaling pathways may indicate on the tumor sensitivity to regorafenib.

Currently regorafenib is approved for treatment of metastatic colorectal cancer (48), advanced gastrointestinal stromal tumors (49), and advanced hepatocellular carcinoma (50), but not approved for sarcomas including UL (51). We found only one published report where regorafenib was used in UL (52). A group of 56 patients with leiomyosarcomas, where 22 patients had UL, showed significantly longer progression-free survival if treated with regorafenib compared to the placebo cohort (52). In addition to leiomyosarcoma, regorafenib improved PFS in synovial sarcoma, but not in liposarcoma (52). When patients from multiple cohorts with non-adipocytic sarcomas were pooled together, median PFS was 4.0 months in the regorafenib arm and 1.0 month in the placebo arm (HR 0.36, P-value <.0001). Median PFS in leiomyosarcoma group was 3.7 months, while our patient did not progress for 10 months.

In this study we also found a new *YWHAE*-*JAZF1* cancer fusion transcript that most probably results in enhanced activity of *JAZF1* moiety. The latter gene controls lipid metabolism by suppressing lipogenesis and increasing lipolysis, and regulates expression of PPARA and PPARD (53, 54). Interestingly, two out of ten the most strongly suppressed molecular pathways in the patient’s tumor were different versions of PPAR signaling pathway which can be a functional consequence of a fusion oncogene activity (**Figure 3A**, bottom). Currently, no meaningful conclusion can be made on possible association of the detected fusion on regorafenib response. Future clinical studies are required to elaborate on that.

Nowadays there are several medical first-generation second opinion platforms that use genetic profiling data like CARIS Molecular Intelligence and Foundation ONE (55–58). Their clinical utility is limited to the analysis of a modest number of clinically actionable mutations and immunohistochemical profiling of a small panel of approved cancer biomarkers. The enclosed targeted panels contain only up to 2% of the total number of protein-coding genes, thus making most part of the cancer exome invisible. Those platforms also don’t use high throughput gene expression data to prioritize therapeutic options in cases when several drugs could be potentially effective. In the present case, the mutation analysis was not informative, whereas it was the Oncobox transcriptomic/molecular pathway profiling that allowed to identify an effective treatment.

Effectiveness of this method was previously published in several case reports (27–30), retrospective (23, 24) and prospective (26, 59) clinical investigations. In this communication, we describe the use of regorafenib, which was selected based on Oncobox analysis of RNAseq data for the treatment of UL with lung metastases after unsuccessful chemotherapy. Regorafenib treatment resulted in a prolonged complete metabolic response and poor yet acceptable toxicity. This case suggests that personalized approach utilizing both mutation and gene expression profiling may be helpful for guiding treatment selection in advanced UL. However, this suggestion is based on an individual case, which is the main limitation of the current study. Larger prospective clinical studies are needed to investigate clinical utility and validity of such an approach. The strength of the current study is the first to our knowledge integrative (WES and RNAseq) prospective analysis of the UL biopsy, which enabled to choose the effective personalized treatment.

## Materials and Methods

The patient provided written informed consent for the analysis of her cancer tissue biosample and for presentation of relevant clinical and molecular data in this paper - for disclosure of sex, histological tumor type, diagnosis, relevant instrumental images, and molecular data including RNA sequencing data and whole-exome sequencing data. The study was conducted in accordance with the Declaration of Helsinki ethical principles. The consent procedure and the design of the study were approved by the local ethical committee of the Medical Holding SM-clinic.

The tumor tissue sample used for gene expression analysis was stored in the form of formalin-fixed paraffin-embedded (FFPE) tissue block at the room temperature. For nucleic acid extraction, we used sections of FFPE block with tumor cell content 95%.

RNA was isolated from FFPE slices and sequenced according to our previous protocols (20, 60). DNA was extracted and used for whole-exome sequencing as described (30). For normalization of gene expression to calculate CNR and pathway activation levels, we used RNA sequencing profiles from ANTE collection for normal tissues (20) of healthy donors killed in road accidents, that was built using the same equipment and protocols.

Gene expression, molecular pathway activation and mutation analyses were performed as described previously (16, 17, 29, 30). For molecular pathway analysis we used previously published database of 3044 molecular pathways involving 9022 human genes (61), but included only pathways with 10 or more genes (n = 1682).

We did Gene Ontology search using GeneOntology tool (http://geneontology.org/) and q-value setting < 0.1 for 20595 genes included in GO terms and verified results using GOrilla software (http://cbl-gorilla.cs.technion.ac.il/) for 19098 HGNC protein coding genes, and using enrichGO clusterProfiler software (org.Hs.eg.db) with ENTREZID as the gene names.

Fusion transcripts were initially screened using STAR-Fusion software. Preliminary files containing fusion candidates were generated and the corresponding RNA sequencing reads were extracted. The output data were manually inspected using UCSC BLAT and UCSC Browser (https://genome.ucsc.edu/) to interrogate fusion candidates according to the following criteria: (*i*) does the read cover exon junction of two different transcripts, (*ii*) if the junction point exactly matches exon termini of known genes with canonic splice sites, (*iii*) if both transcripts are in the same orientation.

## Data Availability Statement

The datasets presented in this study can be found in online repositories. The names of the repository/repositories and accession number(s) can be found below: https://www.ncbi.nlm.nih.gov/bioproject/PRJNA700818.

## Ethics Statement

The studies involving human participants were reviewed and approved by local ethical committee of the Medical Holding SM-clinic. The patients/participants provided their written informed consent to participate in this study. Written informed consent was obtained from the individual(s) for the publication of any potentially identifiable images or data included in this article.

## Author Contributions

AS and AP were involved in patient management and study design. MSu performed molecular analyses. LM analyzed PET-CT data and prepared figures. AG analyzed clinical information. ER, MSo, and AB identified RNA sequencing reads for fusion transcript and characterized it. MZ, AB, and MSo did bioinformatic analysis of RNA sequencing and WES data. AS, ZM, MSo, and AB wrote the manuscript. All authors contributed to the article and approved the submitted version.

## Funding

The study was supported by the Russian Science Fund grant 20-75-10071.

## Conflict of Interest

MSo and AB were employed by OmicsWay Corp.

The remaining authors declare that the research was conducted in the absence of any commercial or financial relationships that could be construed as a potential conflict of interest.

## Publisher’s Note

All claims expressed in this article are solely those of the authors and do not necessarily represent those of their affiliated organizations, or those of the publisher, the editors and the reviewers. Any product that may be evaluated in this article, or claim that may be made by its manufacturer, is not guaranteed or endorsed by the publisher.

## Supplementary Material

Supplementary Figure 1 CT (left) and PET-CT (right) chest scans of the chest. **(A)** A node in S6 of the left lung in March 2019, December 2019 and March 2020; **(B)** A node in S8 of the right lung in March 2019, December 2019 and March 2020.

Supplementary Figure 2 A pathological lesion on the right, with the spread to the area of the iliac vessels (indicated with green arrow), June 2019.

Supplementary Figure 3 Lesions in the right iliac region - secondary altered lymph nodes, with signs of invasion in the right ureter, September 2019.

Supplementary Figure 4 GO visualization of top-30 significant “biological process” terms by R package *enrichplot* (http://bioconductor.org/packages/release/bioc/html/enrichplot.html). All terms passed Benjamini-Hochberg adjusted p-value threshold of 0.05.

Supplementary Figure 5 CT (left) and PET-CT (right) chest scans of the chest in July 2020. **(A)** A node in S6 of the left lung; **(B)** A node in S8 of the right lung.

Supplementary Figure 6 CT (left) and PET-CT (right) scans of the pelvis in July 2020. **(A)** Lesion in the area of the removed right ovary. **(B)** CT and PET-CT scans of the abdomen, axial plane.

Supplementary Table 1 Gene fold changes, Pathway Activation Levels and Balanced Efficiency Scores calculated by the Oncobox method.
