# Supplementary figures and images for: RNA Sequencing for Personalized Treatment of Metastatic Leiomyosarcoma: Case Report
Source: Front Oncol. 2021 Aug 30;11:666001. doi: 10.3389/fonc.2021.666001 (PMC8435728; doi:10.3389/fonc.2021.666001)

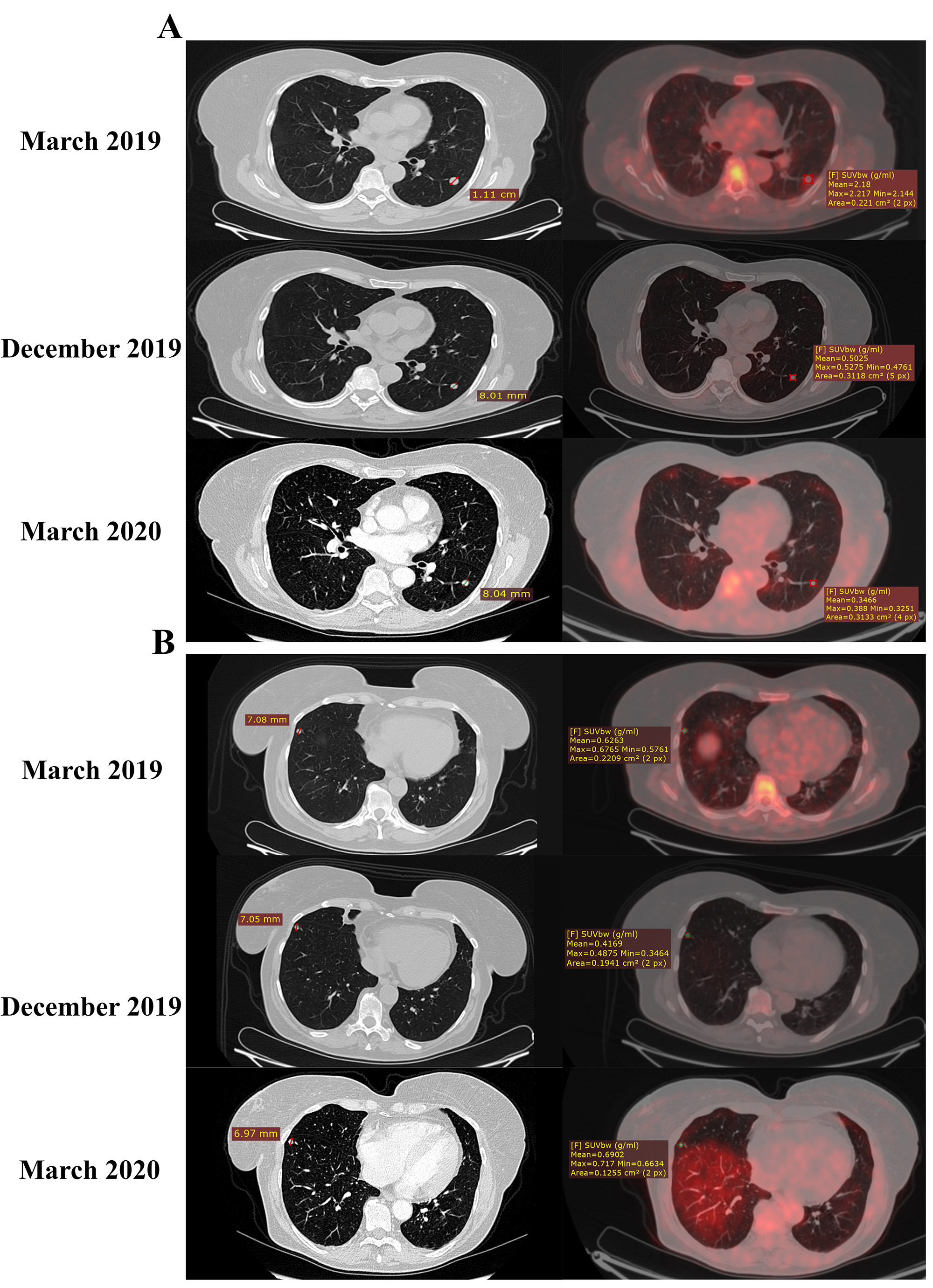

Supplement: Supplementary Figure 1 — CT (left) and PET-CT (right) chest scans of the chest. (A) A node in S6 of the left lung in March 2019, December 2019 and March 2020; (B) A node in S8 of the right lung in March 2019, December 2019 and March 2020. [file Image_1.jpeg]

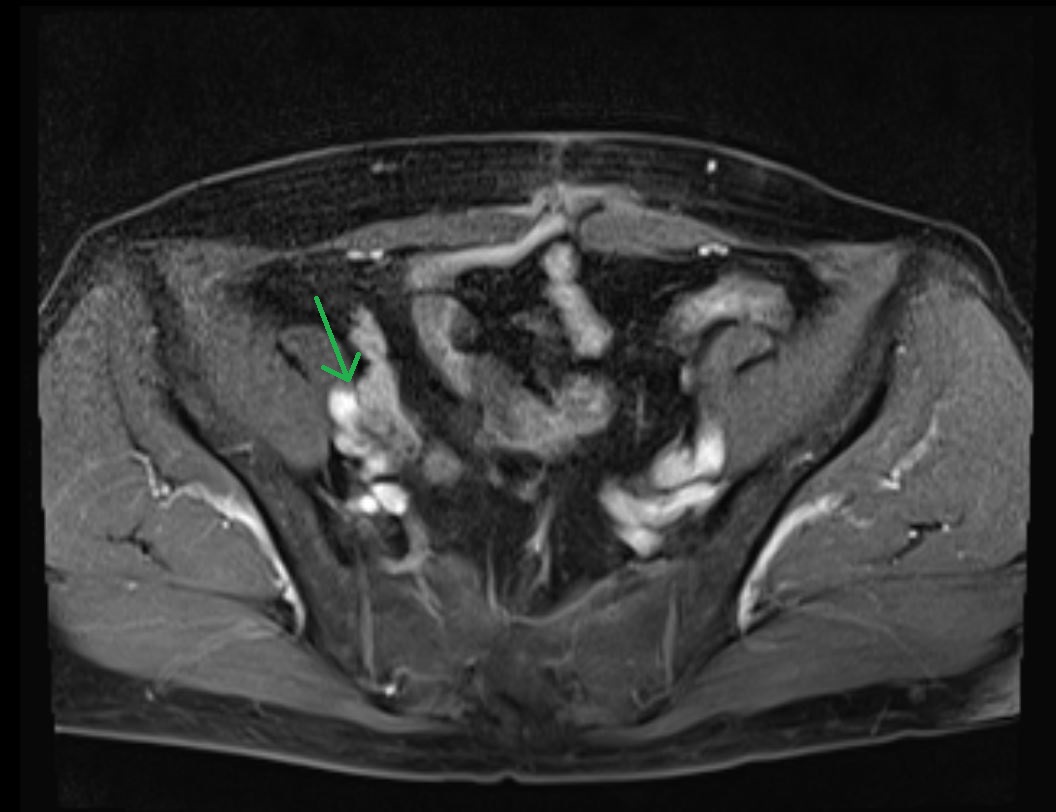

Supplement: Supplementary Figure 2 — A pathological lesion on the right, with the spread to the area of the iliac vessels (indicated with green arrow), June 2019. [file Image_2.jpeg]

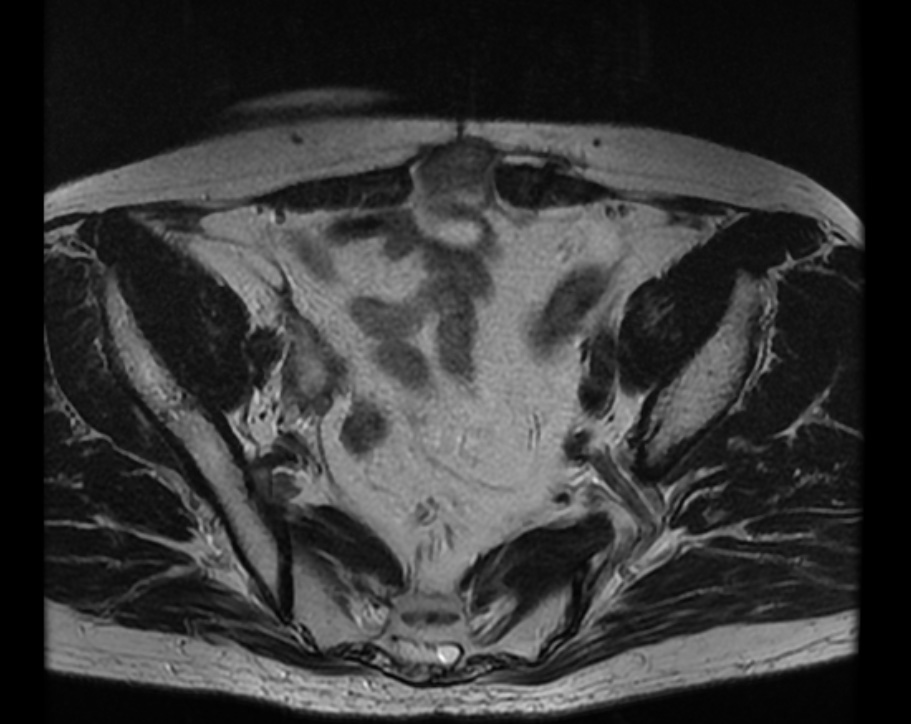

Supplement: Supplementary Figure 3 — Lesions in the right iliac region - secondary altered lymph nodes, with signs of invasion in the right ureter, September 2019. [file Image_3.jpeg]

GO terms for top-100 genes

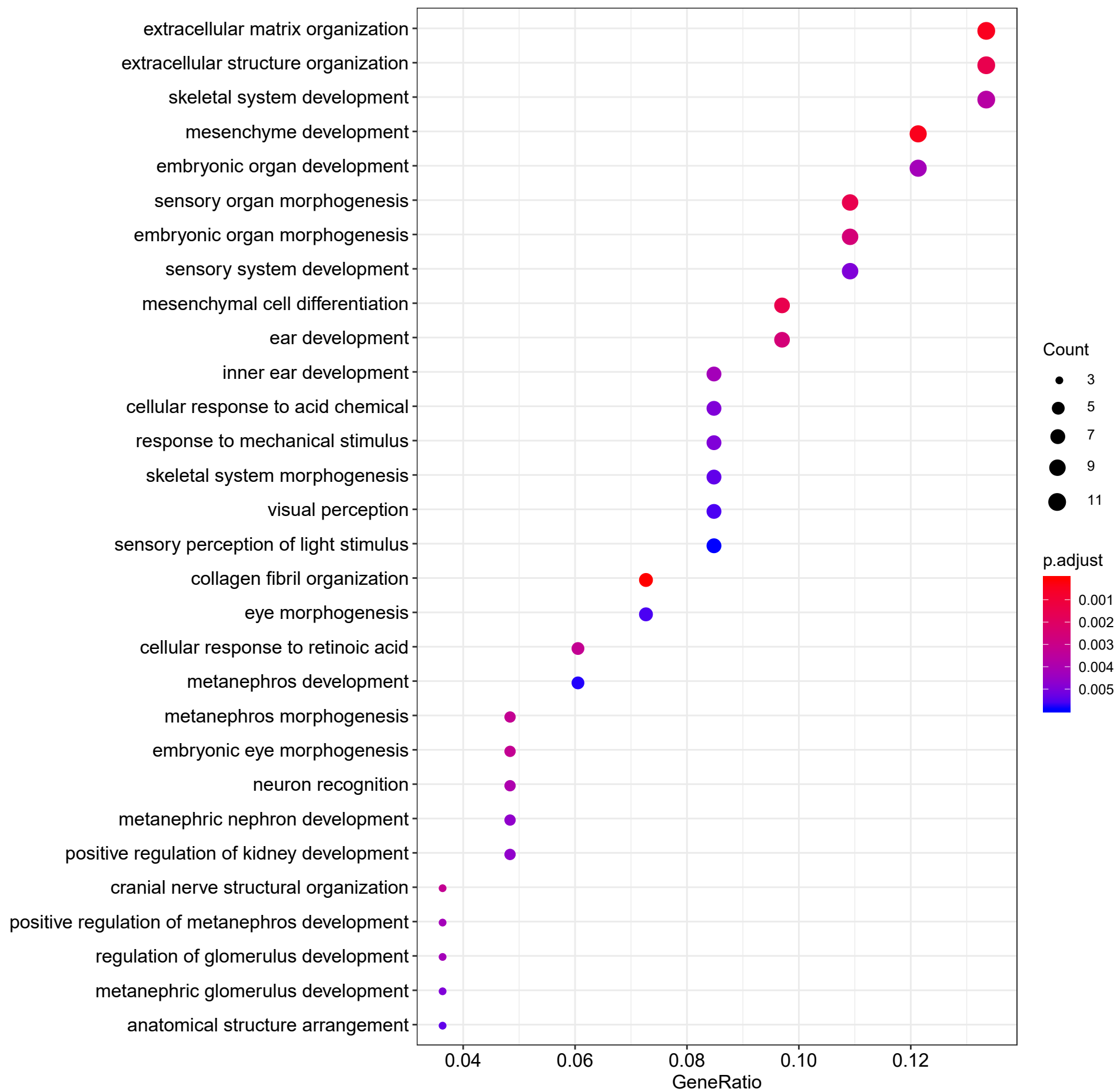

GO terms for bottom-100 genes

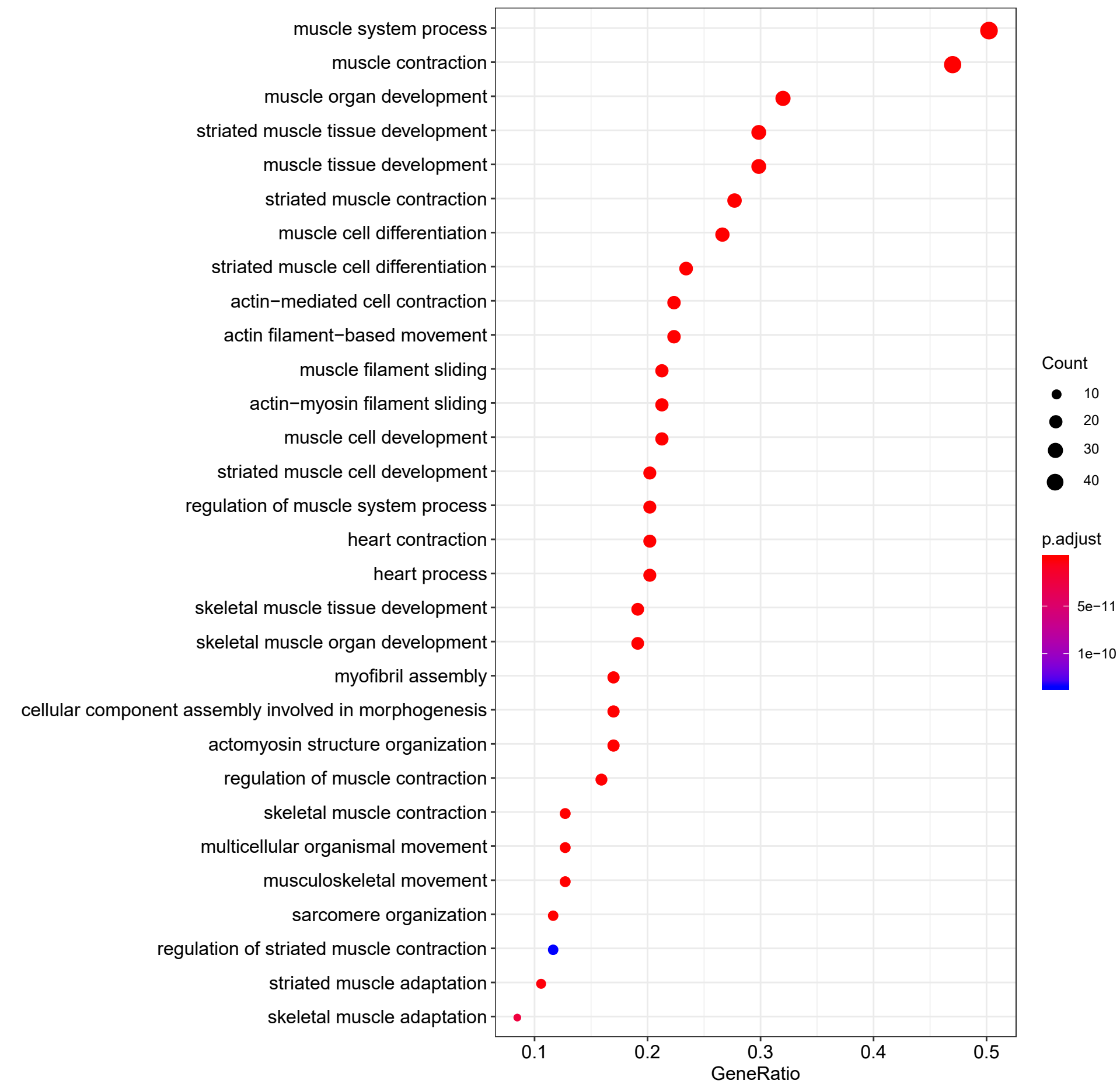

Supplement: Supplementary Figure 4 — GO visualization of top-30 significant “biological process” terms by R package enrichplot (http://bioconductor.org/packages/release/bioc/html/enrichplot.html). All terms passed Benjamini-Hochberg adjusted p-value threshold of 0.05. [file Image_4.pdf]

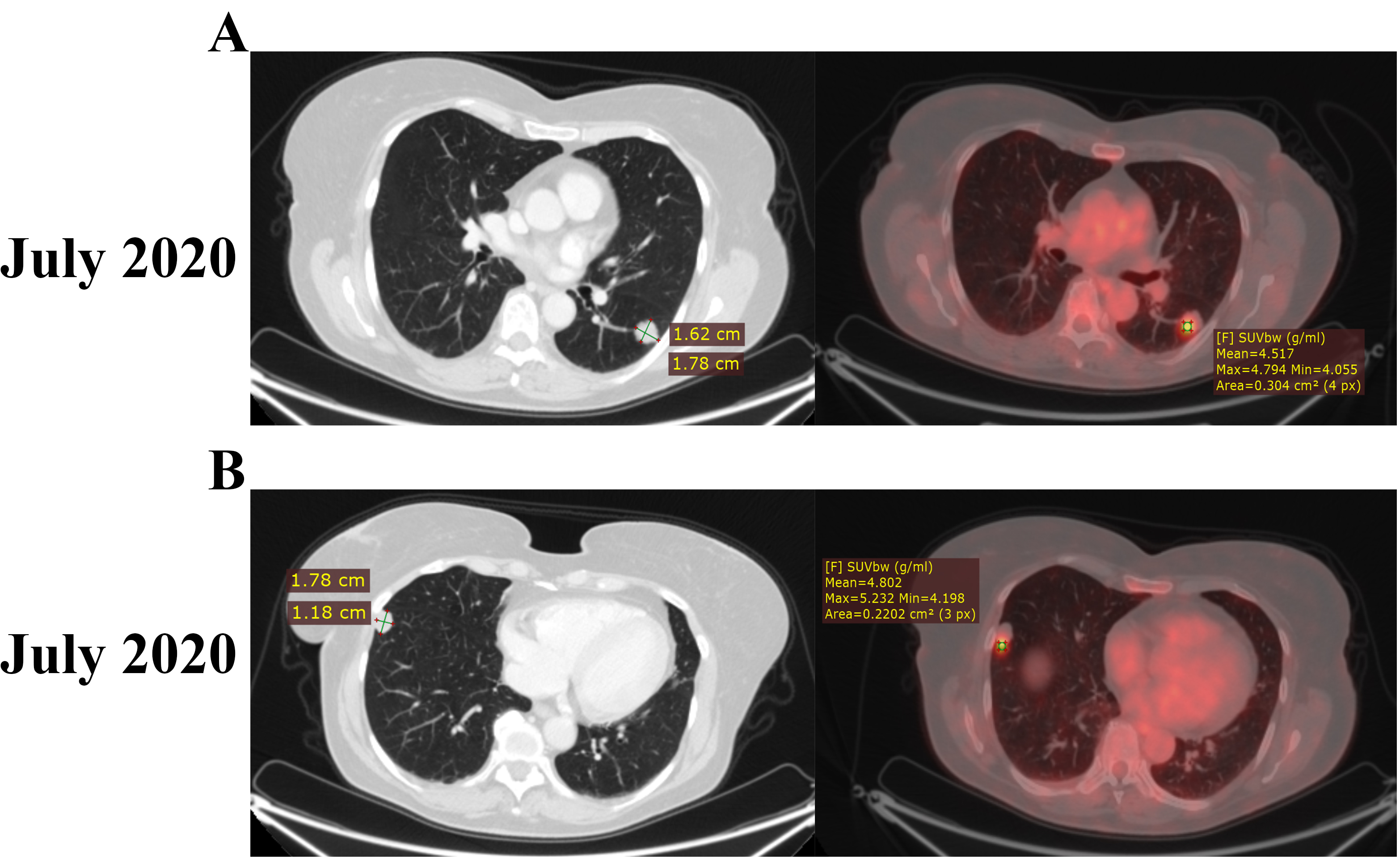

Supplement: Supplementary Figure 5 — CT (left) and PET-CT (right) chest scans of the chest in July 2020. (A) A node in S6 of the left lung; (B) A node in S8 of the right lung. [file Image_5.jpeg]

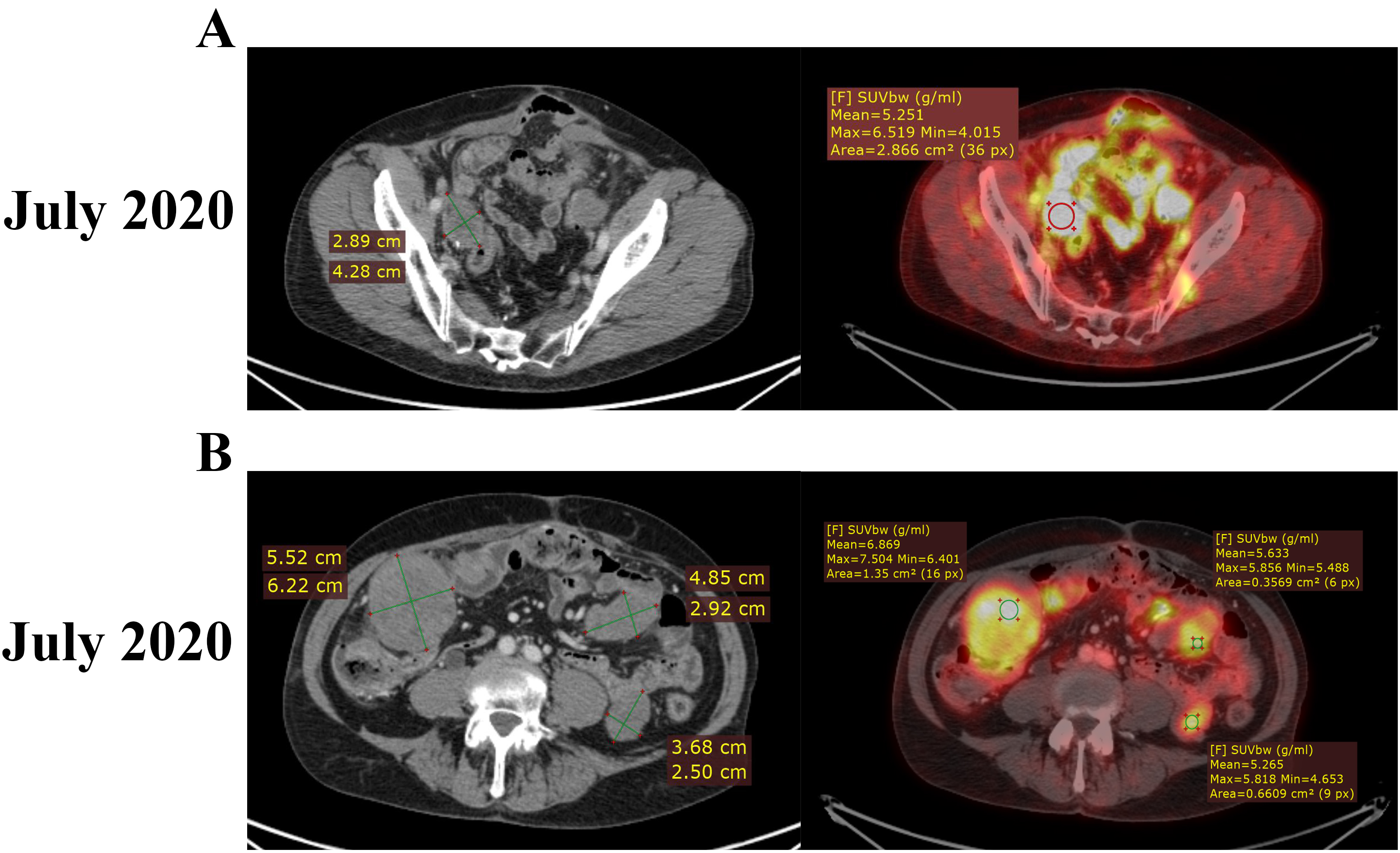

Supplement: Supplementary Figure 6 — CT (left) and PET-CT (right) scans of the pelvis in July 2020. (A) Lesion in the area of the removed right ovary. (B) CT and PET-CT scans of the abdomen, axial plane. [file Image_6.jpeg]
